# Identification of genes involved in serum tolerance in the clinical strain *Cronobacter sakazakii* ES5

**DOI:** 10.1186/1471-2180-13-38

**Published:** 2013-02-15

**Authors:** Sarah Schwizer, Taurai Tasara, Katrin Zurfluh, Roger Stephan, Angelika Lehner

**Affiliations:** 1 Institute for Food Safety and Hygiene, Vetsuisse Faculty, University of Zurich, Zurich, Switzerland

**Keywords:** *Cronobacter sakazakii* ES5, Clinical isolate, Serum tolerance, Tn5-mutagenesis, Identification, PCR, Complementation, Expression analysis

## Abstract

**Background:**

*Cronobacter* spp. are opportunistic pathogens that can cause septicemia and infections of the central nervous system primarily in premature, low-birth weight and/or immune-compromised neonates. Serum resistance is a crucial virulence factor for the development of systemic infections, including bacteremia. It was the aim of the current study to identify genes involved in serum tolerance in a selected *Cronobacter sakazakii* strain of clinical origin.

**Results:**

Screening of 2749 random transposon knock out mutants of a *C. sakazakii* ES 5 library for modified serum tolerance (compared to wild type) revealed 10 mutants showing significantly increased/reduced resistance to serum killing. Identification of the affected sites in mutants displaying reduced serum resistance revealed genes encoding for surface and membrane proteins as well as regulatory elements or chaperones. By this approach, the involvement of the yet undescribed Wzy_C superfamily domain containing coding region in serum tolerance was observed and experimentally confirmed. Additionally, knock out mutants with enhanced serum tolerance were observed. Examination of respective transposon insertion loci revealed regulatory (repressor) elements, coding regions for chaperones and efflux systems as well as the coding region for the protein YbaJ. Real time expression analysis experiments revealed, that knock out of the gene for this protein negatively affects the expression of the *fimA* gene, which is a key structural component of the formation of fimbriae. Fimbriae are structures of high immunogenic potential and it is likely that absence/truncation of the *ybaJ* gene resulted in a non-fimbriated phenotype accounting for the enhanced survival of this mutant in human serum.

**Conclusion:**

By using a transposon knock out approach we were able to identify genes involved in both increased and reduced serum tolerance in *Cronobacter sakazakii* ES5. This study reveals first insights in the complex nature of serum tolerance of *Cronobacter* spp.

## Background

The genus *Cronobacter*, member of the family *Enterobacteriaceae,* comprises seven species – *C. sakazakii*, *C. turicensis*, *C. malonaticus*, *C. muytjensii*, *C. dublinensis*, *C. universalis* and *C. condimenti*[1,2]. They are opportunistic pathogens that can cause septicaemia and infections of the central nervous system primarily in premature, low-birth weight and/or immune-compromised neonates [3]. Most outbreaks have been reported in neonatal intensive care units where the sources of infection have been traced to *Cronobacter* spp. contaminated, reconstituted powdered infant formula (PIF) and/or feeding equipment.

As a foodborne pathogen causing systemic infections, *Cronobacter* spp. must cross the gastrointestinal barrier and, following their tropism for the central nervous system, translocate to and cross the blood–brain barrier (BBB). In that context, it is expected that *Cronobacter* spp. express virulence factors that help in colonization and invasion of mucosal cells [4] as well as effectors that confer the ability of *Cronobacter* spp. to overcome the mechanisms of killing by serum components and/or the human complement system [5,6].

Microbes that cause invasive infections have evolved strategies to protect themselves against the bactericidal action of the serum/complement. Structures of the bacterial cell surface, such as capsules, LPS and outer-membrane proteins have been identified as being responsible for the complement resistance of bacteria [6,7]. For *Cronobacter* spp. it has been shown, that the outer membrane protein Omp A contributes significantly to the survival of the bacteria in the blood [8].

In a more recent study an outer membrane protease Cpa has been identified as a factor that activates plasminogen, thus mediating serum resistance in *C. sakazakii*[9]. However, it has been demonstrated, that there is a considerable degree of variation among *Cronobacter* spp. isolates with respect to their ability to resist serum complement [10]. In a pilot study a set of *Cronobacter* isolates (all species, subspecies) from various origins (clinical, environment, milk powder) was tested for their capacity to survive in human blood and the clinical isolate *Cronobacter sakazakii* ES5 was identified as the most tolerant strain (i.e. ≤ 2 log reduction during incubation in 50% human pooled serum for 120 min) among the *Cronobacter sakazakii* isolates tested (data not shown).

This strain was selected for further experiments aiming for the identification and analysis of genes involved in this feature.

## Results and discussion

### Identification of genes involved in modified serum tolerance in *C. sakazakii*

**Figure 1:**
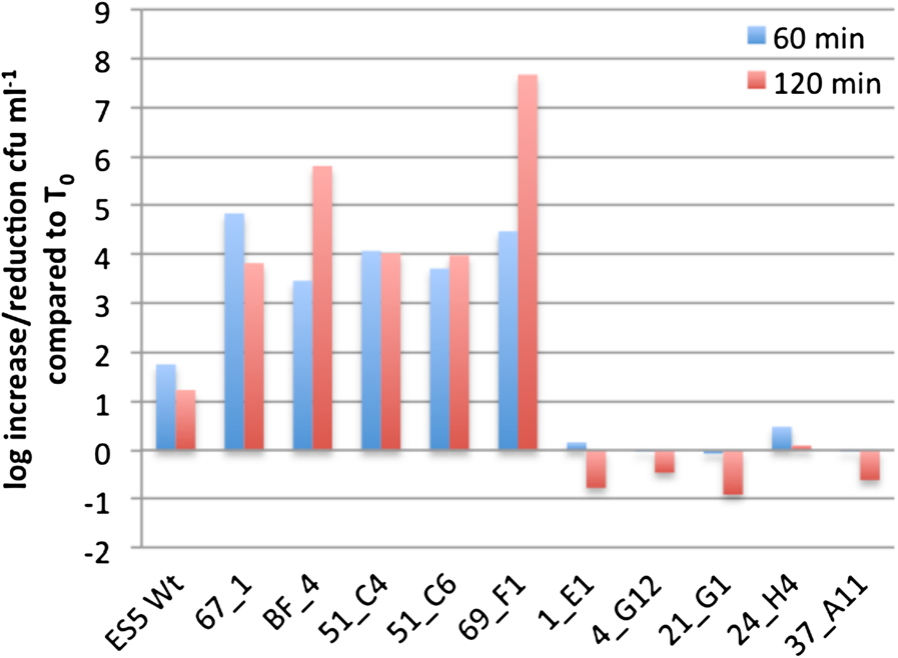
**Sensitivity of *C. sakazakii *ES5 transposon insertion mutants during incubation in 50% HPS for 60 min and 120 min compared to the wt.** Within this graph results are depicted which were generated during the confirmative serum sensitivity tests on mutants selected during the screening procedure in the 96 well format.

Screening of 2749 random mutants from a *C. sakazakii* ES5 Tn5 library for modified serum tolerance revealed 10 candidates for which a significantly increased/reduced tolerance to serum killing (as compared to the wild type) was confirmed. In Figure 1 the variations in the survival of the mutants expressed as log variation (y-axis) over time (x-axis) is depicted. Serum sensitivity was expressed in log variations (number of cfu ml^-1^ after incubation in 50% human pooled serum (HPS) for 60 and 120 min (T_60_, T_120_)/ the number of cfu ml^-1^ of non- serum exposed inoculum (T_0_). By referring the counts after incubation to T_0_, the inoculum variations were corrected for all experiments.

Only mutants for which a single transposon insertion in the chromosome was confirmed were subjected to the subsequent mapping experiments. The sequences obtained were subjected to similarity searches at the NCBI website.

Table 1 summarizes the affected coding regions for the mutants, the closest homologue on the amino acid level and description of the putative function of the protein.

**Table 1:** **Identification and description of affected insertion sites in mutants displaying modified serum resistance in *C. sakazakii *ES5**

	**Annotation**			
**Mutant**	**Phenotype**	**Locus tag closest homologue blastx/organism**	**Protein Name (max ident aa)**	**Description**
67.1^a^	Reduced serum resistance	ESA_04343/*Cronobacter sakazakii* BAA-894	Putative uncharacterized protein (100%)	Putative membrane protein IgaA homolog (*C. turicensis* z3032)
BF4^b^	Reduced serum resistance	ESA_04103/*Cronobacter sakazakii* BAA-894	Putative uncharacterized protein (100%)	Hypothetical protein, conserved domain: Wzy_C superfamily O-antigene ligase
51_C4^c^	Reduced serum resistance	ESA_03258/*Cronobacter sakazakii*BAA-894	DNA binding transcriptional regulator FruR (99%)	Fructose repressor
51_C6^c^	Reduced serum resistance	CSE899_07155/*Cronobacter sakazakii* E899	Hypothetical protein (100%)	FadR, GNTR family of transcriptional regulator, winged helix-turn helix DNA binding domain.
69_F1^c^	Reduced serum resistance	ESA_01368 *Cronobacter sakazakii* BAA-894	Hypothetical protein (98%)	DnaJ domain protein
1_E1^c^	Increased serum resistance	CSE899_13864 *Cronobacter sakazakii* E899	Copper homeostasis protein CutC (100%)	Uncharacterized protein involved in copper resistance
4_G12^c^	Increased serum resistance	ESA_03283 *Cronobacter sakazakii* ATCC BAA-894	Hypothetical protein (99%)	DjlA
21_G1^c^	Increased serum resistance	ESA_02809/*Cronobacter sakazakii* BAA-894	Hypothetical protein (99%)	Hha toxicity attenuator, YbaJ “biofilm formation regulator” *C. sakazakii* E899
24_H4^c^	Increased serum resistance	ESA_03832/*Cronobacter sakazakii* BAA-894	Hypothetical protein (100%)	ribonuclease activity regulator protein RraA
37_A11^c^	Increased serum resistance	Ctu_3p00270/ *Cronobacter turicensis* z3032	Hypothetical protein (99%)	On Plasmid pCtu3 of *C. turicensis* z3032 – no annotation available

^a^ obtained from the study by Johler et al., 2010 [11].

^b^ obtained from the study by Hartmann et al., 2010 [13].

^c^ this study.

Identification of the respective mutated sites from mutants displaying reduced serum resistance included genes coding for surface and membrane proteins (67.1, BF4), (transcription) regulatory genes (51_C4, 51_C6) as well as a DnaJ domain containing protein (69_F1). Mutant 67_1 represents a knock out in the *igaA* coding gene. This non-pigmented mutant has been identified in the study by Johler et al. (2012) but was not subject of further investigation in this study [11]. However, this protein was identified in *Salmonella* Typhimurium as a membrane protein that attenuates the response of the RcsCDB signalling system to environmental stress. The Rcs two component system is known to be involved in the (positive/negative) regulation of a number of target genes including biofilm formation and pathogenicity. Thus, it has been reported, that the constitutive activation of this system dramatically attenuates *Salmonella* virulence [12].

Mutant BF4 was originally described in the study by Hartmann et al. (2010) where it was found to produce less biofilm on polystyrene [13]. The transposon insertion affected a site with 100% homology to the locus ESA_04103 of the *C. sakazakii* ATCC BAA 894 genome (CP000783.1) to which the annotation hypothetical protein was available at that time. However, BLASTx analysis of the respective protein reveals homology to proteins containing a conserved Wzy_C superfamily domain. The coding region for this protein must not be confused with the gene for the Wzy protein which is part of the O- antigen gene locus (often referred to as *rfb* locus in *Enterobacteriaceae*) located between ESA_01177 and ESA_01190 the function of which is annotated as O-antigen polymerase. The O-antigen forms part of the lipopolysaccharide (LPS) in the outer membrane of Gram-negative bacteria and is one of the most variable constituents on the cell surface. There are currently seven (O1-O7) different O-antigen serotypes described for *C. sakazakii* and the putative organization of the genes included in the different clusters has been published recently [14,15]. As in one of these serotypes (O7), the *wzy* gene does not seem to be part of the cluster it has been proposed, that a different, yet unknown gene mapping elsewhere in the chromosome may code for this essential function and we further hypothesized that the ESA_04103 coding region may have been a candidate for this. However, determination of the O-antigen serotype of the *C. sakazakii* ES5 strain by application of a recently developed PCR based serotyping scheme [16] revealed that this strain belongs to the O2 serotype (data not shown).

**Figure 2:**
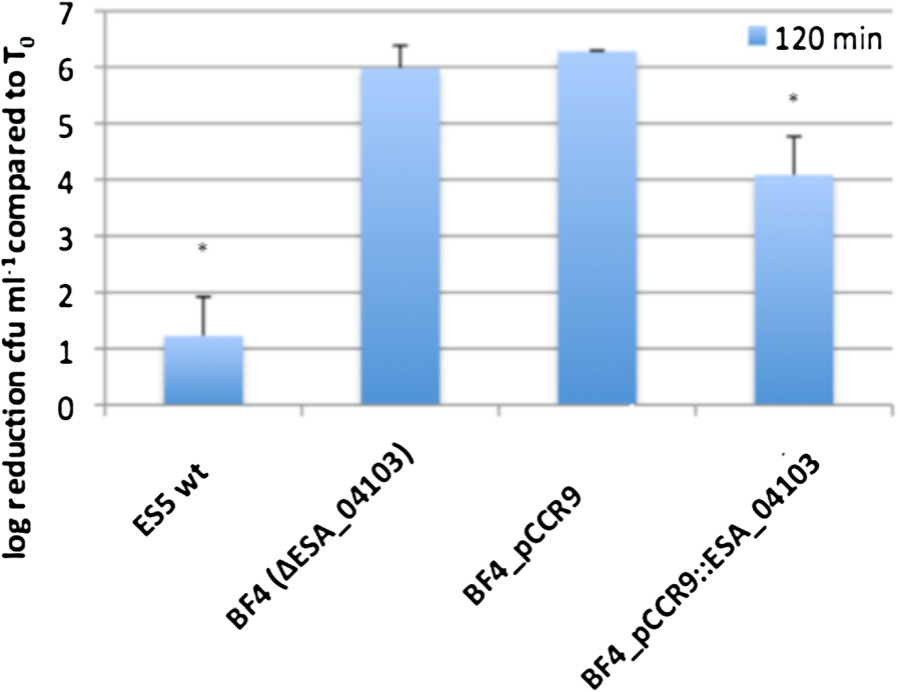
**Serum sensitivity test on *C. sakazakii *ES5 wt, mutant BF4 (ΔESA_04103), mutant containing the empty vector (BF4_pCCR9) and mutant complemented with the intact ESA_04103 gene (BF4_pCCR9::ESA_04103) after incubation in 50% HPS for 120 min (T**_**120**_**).** The means and standard deviations (±1SD) from two independent experiments are presented. An asterisk above the bars indicate statistically significant differences.

As to date there is no experimental data on the involvement of the ESA_04103 coding region in serum tolerance available, we aimed to investigate whether an intact copy of the ESA_04103 gene provided in trans to the ESA_04103 deleted mutant BF4 would restore the serum tolerance to wild type level. Therefore this gene including its putative native promoter region was cloned onto a low copy expression vector and the resulting construct was transformed into BF4 mutant. Serum sensitivity tests were performed using the *C. sakazakii* ES5 wt strain, the BF4 (ΔESA_04103) mutant, the BF4 (ΔESA_04103) mutant containing an empty pCCR9 vector (BF4_pCCR9) and the complemented mutant BF4_pCCR9::ESA_04103*.* The results of these experiments are depicted in Figure 2. An inactivation around 5 log during incubation in 50% human serum for 120 min was observed in the BF4 (ΔESA_04103) mutant as well as the mutant containing the low copy vector pCCR9, whereas the survival of the mutant with supplied vector pCCR9 and ESA_04103 was restored to 4 log reduction cfu ml^-1^ compared to T_0_ compared to the wt with 1.2 log reduction. We could, however, not completely restore the serum survival to wild type levels in the complemented mutant. This may be explained (in part) by the unknown copy number of the mRNA for this gene in the wild type during incubation in serum and/or by possible polar effects.

Mutant 69_F1 was identified to be affected in a gene coding for a DnaJ domain family protein. Members of this family are essential for their interaction with DnaK chaperone and activation of its ATPase activity. In *Edwardsiella tarda* it was recently demonstrated that DnaJ and DnaK play a crucial role in general bacterial virulence, in blood dissemination capacity [16].

Interestingly, by using the Tn5 approach we found an equally high number of knock out mutants, that showed an enhanced survival in human serum compared to the wild type. One of the obvious possibilities to explain this phenomenon would be the knock out of regulatory elements (repressors) which would lead to a subsequent activation/constitutive expression of the respective phenotype. Mutant 24_H4 (Δ*rraA*) may fall into this category. The region affected by the transposon in this mutant shows homology to the ribonuclease regulator protein RraA. This protein acts as an inhibitor of the essential endoribonuclease RNase E, which itself plays a crucial role in global mRNA metabolism as well as in the maturation of functional RNAs such as rRNAs, tRNAs, tmRNA, and small regulatory RNAs [17-20]. However, Lee et al. [21] demonstrated that ectopic expression of RraA itself affects the abundance of more than 700 transcripts in *Escherichia coli*, thus making it difficult to hypothesize upon the influence of the knock out of this gene on the enhanced survival of the mutant in human serum.

More surprising was the finding that deletions in genes putatively coding for (co-)chaperones lead to an enhanced survival in human serum. One of those, namely 4_G12 (Δ*djlA*), is a member of the J-domain protein family. DjlA can substitute for DnaJ co-chaperone [22] and seems to have multiple functions. However, it has also been described that DjlA negatively regulates the response of the two component RcsCDB signaling system to envelope stress. The Rcs signal transduction system positively regulates the expression of many different genes among those are the ones forming the capsular polysaccharide synthesis operon *(cps)*[23]. The expression of capsules may provide protection from serum killing components (see above). In a study by Shiba et al. [24] it was demonstrated that *djl*A deletion resulted in increased activation of the Rcs system. This might positively regulate *cps* transcription.

Mutant 21_G1 (Δ*ybaJ*) exhibiting an enhanced serum tolerance was shown to be affected in a gene coding for the YbaJ protein. It has been proposed that YbaJ and its adjacent protein Hha may form a so called toxin-antitoxin pair where YbaJ (antitoxin) negatively regulates the expression of Hha (toxin), the latter one (among other functions) serving as a repressor for type 1 fimbriae [25]. Type 1 fimbriae are highly immunogenic, thus a strain not expressing these structures may have an advantage in survival during exposure in human serum [26].

**Figure 3:**
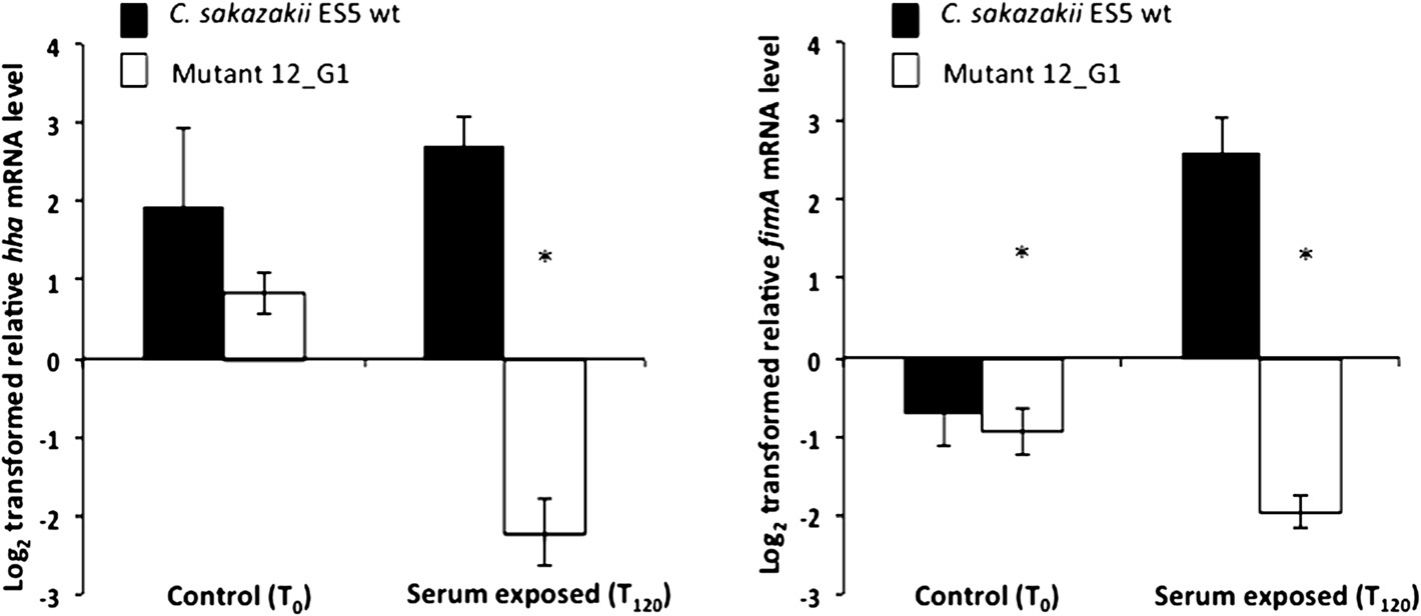
**Relative levels of *hha *and *fimA *mRNA in control (T**_**0**_**) and serum treated (T**_**120**_**) *C. sakazakii *ES5 wt and mutant 21_G1 (Δ*ybaJ*) cells.** RNA was isolated from mid exponential growth stage cells prior (T_0_) and after (T_120_) human serum exposure. Values were normalized using 16S rRNA as a reference gene. The means and standard deviations (±1SD) from three independent experiments are presented. An asterisk above the bars indicate statistically significant differences in mRNA levels between the *C. sakazakii* ES5 wt and mutant (P < 0.05).

In the present study we further examined the hypothesis that the disruption of the regulatory gene *ybaJ* may lead to an activation of the Hha protein which in turn would negatively influence transcription of the key fimbrial structural gene *fimA.* RT-qPCR experiments were performed in order to quantify *hha* and *fimA* mRNA levels in the *C. sakazakii* ES5 wt and mutant 21_G1(Δ*ybaJ*) strains, before and after exposure to human serum. The levels of *fimA* mRNA were more than 4.5 log lower in the mutant 21_G1(Δ*ybaJ*) strain compared to the *C. sakazakii* ES5 wt strain. The *hha* mRNA levels were for the mutant compared to the wt 5 log lower and not like expected higher, suggesting that the deletion of the *ybaJ* gene did not result directly in a de-repression/ activation of the *hha* gene in our experimental set up (Figure 3). Our results rather suggest that *ybaJ* itself may be involved in the regulation/activation of the expression of the type 1 fimbriae in *C. sakazakii*.

## Conclusions

By using a transposon knock out approach we were able to identify structural and regulatory genes in *Cronobacter sakazakii* ES5, deletion of which resulted in a dramatically reduced capability to survive in serum. Additionally, several mutants were found displaying an enhanced survival in serum as compared to the wild type. Analysis of the genetic elements possibly responsible for this phenotype revealed genes coding for chaperone-like proteins, regulatory (repressor) elements as well as genes for structures or components representing immunogenic targets. The deletion of the *ybaJ* element which is part of the antitoxin-toxin pair YbaJ-Hha resulted in an abolished expression of a key element of the type 1 fimbriae. The absence of the latter most likely accounted for the enhanced survival of this mutant in human serum.

## Methods

### Bacterial strains and culture conditions

*Cronobacter sakazakii* strain E5, a clinical strain was used in this study. Wild type and mutant strains, *E. coli* DH5 alpha as well as plasmids and primers that were included and constructed during the transposon library screening, the mutant complementation (BF4) and the expression (21_G1) experiments are summarized in Table 2. All strains were incubated at 37°C in Luria–Bertani (LB) broth, over night with gentle shaking. When appropriate, antibiotics were used at the following concentrations: kanamycin at 50 μg ml^-1^ and tetracyclin at 50 μg ml^-1^.

**Table 2:** Material used in this study

**Strains/plasmids/primers**	**Genotype/characteristic(s)/sequences**	**Source or reference**
**Strains**		
*Cronobacter sakazakii*		
ES5 (wild type)	Human isolate	Hartmann et al., 2010, Johler et al., 2010 [11,13]
BF4 (mutant)	ΔESA_04103*,* Kan^R^	Hartmann et al., 2010 [13]
BF4_pCCR9	BF4 harboring pCCR9, Kan^R^, Tet^R^	This study
BF4_pCCR9::ESA_04103	BF4 harboring pCCR9:: ESA_04103, Kan^R^, Tet^R^	This study
21_G1 (mutant)	Δ*yba*J*,* Kan^R^	This study
*Escherichia coli* DH5 alpha	F– Φ 80*lac*ZΔM15 Δ(*lac*ZYA-*arg*F) U169 *rec*A1 *end*A1 *hsd*R17 (rK–, mK+) *pho*A *sup*E44 λ– *thi*-1 *gyr*A96 *rel*A1	Epicentre
Plasmids		
pUC19	High copy cloning/expression vector Amp^R^	Epicentre
pCCR9	Low copy cloning/expression vector, Tet^R^	Randegger et al., 2000 [28]
pCCR9::ESA_04103	pCCR9::ESA_04103, Tet^R^	This study
Primer for sequencing		
KAN-2FP1	5^′^-ACC TAC AAC AAA GCT CTCATC AAC C-3^′^	Epicentre
pCCR9-F	5^′^-TTT GAC AGC TTA TCA TCG-3	This study
pCCR9-R	5^′^-CCT ATG GAA GTT GAT CAG-3	This study
Primer for complementation		
BF4f^1^	5^′^-GAC GCC **AAG CTT**GCG CGA GCC TGC GTT TAA-3^′^	This study
BF4r^2^	5^′^- AGT CTG **GGA TCC**AAA CAT TAT CCT TCT TTA TAG-3^′^	This study
Target/primer for expression		
ybaJf	5^′^-CGG CAT GAT ATA GCG CAG-3^′^	This study
ybaJr	5^′^-GAT GTG TAT AAG AGA CAG-3^′^	This study
hhaf	5^′^-CAA ACC ATT AAC CAA AAC CG-3^′^	This study
hhar	5^′^-CGG AAT TTT ATC GTA GAG CTT-3^′^	This study
fimAf	5^′^-AAA CCG CGT TTA CTG G-3^′^	This study
fimAr	5^′^-GCA ACG GAG TTT GCT T-3^′^	This study
16S rRNAf	5^′^-GTG TTG TGA AAT GTT GGG T-3^′^	This study
16S rRNAr	5^′^-ACT AGC GAT TCC GAC TT-3^′^	This study

^1^ Primer sequence is underlined, recognition site for restriction enzyme *Hind* III is given in bold.

^2^ Primer sequence is underlined, recognition site for restriction enzyme *Bam* HI is given in bold.

### Identifcation of transposon mutants modulating serum tolerance in *Cronobacter sakazakii* ES 5

A random transposon mutant (EZ-Tn5 **<** KAN-2 **>** Tnp) library of the clinical isolate *Cronobacter sakazakii* ES5 [11,13] was screened for modified (i.e. significant log variation in survival during exposure compared to wild type) survival in 50% human pooled serum (HPS) over a period of 120 min. For these experiments, the mutants were grown in 96 well microtiterplates overnight in LB supplemented with 50 μg/ml kanamycin at 37°C. Ten μl of these overnight cultures were transferred into a 96 well screening plate containing 50 μl HPS and 40 μl 0.9% NaCl per well and incubated for 120 min at 37°C (T_120_). Concentrations of bacterial cultures were determined by OD_590nm_ measurement at T_0_ and T_120_ and compared to respective wild type measurements. Thresholds of (1) more than 2 times reduction and (2) more than 7 times increase of OD value during incubation for 120 min relative to the wild type values were set in order to identify potential candidates which were subsequently subjected to a confirming serum sensitivity test.

### Confirmative serum sensitivity tests

LB grown overnight cultures were diluted 1:20 in 10 ml LB and allowed to grow at 37°C to OD_590nm_ = 0.5. Cells were washed twice in 0.9% NaCl, resuspended in 5 ml 0.9% NaCl and diluted to 10^-2^. These dilutions (= 10^0^) served as inoculum for the experiments in 50% human serum. Concentrations of bacterial inoculations at T_0_ were determined by plating 100 ul of 10^-3^, 10^-4^ and 10^-5^ dilutions of the inoculum on LB plates and enumeration of CFU after incubation at 37°C overnight. Two hundred fifty μl HPS was mixed with 50 μl of the above mentioned dilution (10^0^, approx. 10^6^ CFU ml^-1^) and 200 μl of 0.9% NaCl and incubated at 37°C. Survival of the bacterial cells during incubation in 50% HPS was followed by plate count enumeration (plating of 100 ul of a dilution series 10^-1^ – 10^-5^) after 60 and 120 min (T_60_, T_120_). Sensitivity during exposure was expressed in log reduction rates as number of bacteria that survived treatment/number of bacteria in non – serum- exposed inoculum = T_0_). The activity of the human pooled serum (HPS) used for the experiments was tested by comparing cfu ml^-1^ determined after incubation of *C. sakazakii* E5 strain in 50% native or heat inactivated (56°C for 30 min) HPS for 120 min for each new batch (batch control, data not shown).

During serum sensitivity tests on (pCCR9 vector containing) mutants BF4_pCCR9 and BF4_pCCR9::ESA_04103, the serum as well as the plate count medium for enumeration was supplemented with kanamycin and tetracycline both at 50 μg ml^-1^.

### Identification of transposon insertion sites

All kits for DNA isolation and purification were obtained from Qiagen (Hilden, Germany) and handled by following the manufacturer’s instructions. Unless otherwise stated, chromosomal DNA was isolated using the DNeasy Blood and Tissue kit. Plasmids were extracted with the QIAprep Spin Miniprep or Plasmid Midi kits. DNA fragments from PCRs, restriction digests, and agarose gels were purified using the MinElute PCR Cleanup kit and the MinElute Gel Purification kit, respectively. The concentration of nucleic acids was determined using a Nanodrop ND-1000 UV/Vis spectrophotometer (NanoDrop Technologies, Wilmington, DE). Mutants with confirmed phenotype were further subjected to Southern blot analysis in order to determine the chromosomal transposon copy number [11]. Only mutants for which a transposon copy number of one was confirmed were subject of further analysis. Mapping of transposon insertion sites using a subcloning approach was performed as described previously [11]. In brief, chromosomal DNA of the transposon mutants was digested with *SphI*. The fragments were ligated into pUC19 (Table 2) digested with the same enzyme. After ligation (12 h at 16°C) the construct was electroporated into *E. coli* DH5 alpha (Table 2). Transformants carrying a plasmid containing the transposon (= kanamycin cassette) were identified by plating the transformants on LB supplemented with kanamycin. Plasmids were extracted from the selected clones, and the transposon-flanking regions were sequenced with primer KAN-2 FP1 (Table 2). Transposon insertion sites were determined by sequencing the junctions between the Tn5 transposon sites and the ES5 chromosomal DNA. All sequencing was outsourced (Microsynth, Balgach, Switzerland). The sequences obtained from each mutant were determined by similarity search using BLASTn and BLASTx at the NCBI website http://blast.ncbi.nlm.nih.gov/Blast.cgi[27]. The original nucleotide sequences obtained for the mutants after sequencing are provided as supplementary data (Additional file 1).

The cloning, restriction enzyme analysis, and transformation of *C. sakazakii* were performed using standard techniques. Enzymes and respective buffers were obtained from Roche (Basel, Switzerland) or New England Biolabs (Ipswich, MA).

### Complementation experiment with serum sensitive mutant and BF4 (ΔESA_04103)

The ESA_04103 locus was amplified using primer pair BF4f and BF4r (Table 2). This primer pair was designed based on the whole genome sequence of *Cronobacter sakazakii* BAA-894 (CP000783.1) spanning the region from 4058124 to 4059648, including the putative coding sequence as well as 220 bp upstream of the open reading frame in order to ensure the inclusion of the native promoter. The amplification mix contained 0.4 μM of primers, 1 x AccuPrime (Invitrogen) buffer 2 (60 mM Tris-SO_4_ (pH 8.9), 18 mM (NH_4_)_2_SO_4_, 2 mM MgSO_4_, 2 mM dGTP, 0.2 mM dATP, 0.2 mM dTTP, 0.2 mM dCTP, thermostable AccuPrimeTM protein, 1% glycerol) and 2 U AccuPrime Taq DNA Polymerase High Fidelity (Invitrogen). Following PCR conditions were used: 94°C for 30 s followed by 35 cycles of 94°C for 30 s, 54°C for 30 s and 68°C for 120 s.

The resulting PCR products were double digested with the restriction enzymes *Hind* III and *Bam* HI and cloned into the low copy vector pCCR9 [28] which had been digested with the respective enzymes to create the complementation vector pCCR9::ESA_04103. The construct was transformed into the BF4 mutant strain by electroporation and transformants were selected on LB agar supplemented with kanamycin and tetracycline. The correct insertion of the desired fragment was confirmed by amplification and sequencing of the insert of a complemented BF4 mutant using primers located on the pCCR9 vector (pCCR9-F and pCCR9-R, Table 2) and employing the conditions as described during the complementation cloning approach. The sequence of the insert is provided in Additional file 1. Additionally a BF4 mutant containing the pCCR9 vector (BF4_pCCR9) only (no insert) was created and used together with the complemented strain BF4_pCCR9::ESA_04103 in the serum sensitivity assay as described above. The serum assays were carried out in duplicates (= two independent experiments).

### Serum exposure and RNA purification

An 0.5 ml aliquot of a stationary phase grown culture of the wt and mutant strain was used to inoculate 10 ml of LB and grown to the mid exponential growth stage (OD_590nm_ = 0.5) at 37°C. *Cronobacter* cells were washed twice in 10 ml and finally resuspended in 5 ml of 0.9% NaCl solution. Two and half milliliters of the resuspended *Cronobacter* cells were mixed with 12.5 ml HPS and 10 ml 0.9% NaCl. Aliquots of 10 ml were promptly collected. The mixtures were incubated for 120 minutes at 37°C and a second set of aliquots was collected. RNA profiles in collected aliquots were promptly preserved using the bacterial RNA Protect Reagent (Qiagen). *Cronobacter* cell pellets were immediately processed or frozen at −70°C for total RNA extraction at a later stage. Total RNA was isolated using the Qiagen RNeasy Plus Mini kit (Qiagen) with minor modifications to the original kit protocol. *Cronobacter* cells resuspended in 0.5 ml RNeasy Plus Mini Kit lysis buffer (Qiagen) were transferred on to the lysing bead matrix in MagNA lyser tubes and mechanically disrupted in the MagNA Lyser Instrument (Roche Molecular Diagnostics). Two DNA removal steps were incorporated by using a genomic DNA binding column included in the RNeasy Plus Mini Kit as well as by performing an in-column DNAseI (RNase-Free DNase; Qiagen) digestion of the samples bound to the RNA spin column. Total RNA was eluted from the column into 30 μl of RNAse-free water. RNA yields were determined using the Nanodrop ND-1000 spectrophotometer (Nano Drop Technologies, Wilmington, DE). RNA quality was assessed using the Agilent 2100 Bioanalyzer (Agilent Technologies, Santa Clara, CA, USA). Purified RNA was immediately frozen −70°C for long-term storage.

### DNA synthesis and quantitative real time PCR

The synthesis of cDNA was performed using the Quantitect Reverse Transcription Kit (Qiagen). One microgram of total RNA was reverse transcribed to cDNA in 20 μl. Generated cDNA was amplified by quantitative real-time PCR using the Light Cycler 480 instrument (Roche Molecular Diagnostics, Rotkreuz, Switzerland). Primers used for the amplification of the target (*hha* and *fimA*) and reference (16S rRNA) genes are listed in Table 2. Primers were designed using the LC probe design software (Roche Molecular Diagnostics, Penzburg, Germany). Quantitative real-time PCR mixtures contained Light Cycler R 480 SYBR Green I Master (5 μl), forward and reverse primer mixture (2.5 μl) and 100 ng of the cDNA template (2.5 μl). The PCR cycling conditions were as previously described [29]. Reference gene validation was performed as previously described [30], and this established that 16S rRNA mRNA levels were suitable for normalization of relative mRNA quantification under experimental conditions of the present study. The *hha* and *fimA* mRNA levels were quantified relative to the 16S rRNA reference gene and the Light Cycler 480 Relative Quantification Software (Roche Molecular Diagnostics). The relative *hha* and *fimA* mRNA levels obtained after normalization were log converted and data shown are based on the means and standard deviations from three independent assays. The statistical significance of differences in *hha* and *fimA* mRNA levels between *Cronobacter* wt and mutant strains were analyzed using t-tests, and P-values <0.05 were considered to be statistically significant.

## Competing interests

The authors declare that they have no competing interests.

## Authors’ contributions

SS carried out the majority of the experiments, KZ helped during the molecular work. AL and TT conceived the study design, coordinated the molecular work and helped to draft the manuscript. TT contributed to the interpretation of the RT PCR data. RS participated in the design of the study and helped to draft the manuscript. All authors read and approved the final manuscript.

## Supplementary Material

Additional file 1 Results of the sequencing of the transposon insertion flanking sites of the mutants identified in this study, B: Sequence of the ESA_04103 insert after amplification of the pCCR9::ESA_04103 complemented BF4 mutant. Click here for file
