# Supplementary material for: Identification of genes involved in serum tolerance in the clinical strain Cronobacter sakazakii ES5
Source: BMC Microbiol. 2013 Feb 15;13:38. doi: 10.1186/1471-2180-13-38 (PMC3621496; doi:10.1186/1471-2180-13-38)
Supplement: Additional file 1 — Results of the sequencing of the transposon insertion flanking sites of the mutants identified in this study, B: Sequence of the ESA_04103 insert after amplification of the pCCR9::ESA_04103 complemented BF4 mutant. [file 1471-2180-13-38-S1.pdf]

Supplementary file 1: Sequencing results of the flanking regions of the transposon insertion sites of all mutants analysed in this study. Sequencing result of a BF4 transformant (insert in pCCR9) used in the complementation experiment.

67.1
NTCGNGATGGTTGAGANGTGTATAAGAGACAGGAATTNCTTCNATACGGGTGCGGCTGC
GCTGCNGCGGCGCAGCGCGNCGACGCCGTTATACAGCGCACAGATGAGCATCACCANCA
TCAGGAGTGTGGTGCCGAAATAGCGCCACAGCCCGGCGCTGTCAGGGATACTGCTGAGC
GTNACATGCTGGGTGCCGTTGGCGTCGGTGCGCAGGCTGGTAACGACGCCTTCCGCATGG
AATGGCGTCTGCNTCANCATACCCGCCAGCCGCTGGAACTCTTTCCACTGCTCCTGCGGC
GGCNAATCATACNGCGACGNCNGCGGCAGTGGCTGGTTCACCATATCTTTGNCTTNATCG
NTGATAATCATAAAGCCGCCCGGTGGCGGGCTGTTGAGCGACTGCNCGGCGCGGCTGGN
TTCACGTACGAAGAACGGCGCGGTGGAAGCAATCACCAGATTATCGAGCGATTCANCGC
TGACCGGGCGCANCAGGACNTTAATGCCGTCGAGCTTGCCTTCGCTGGCGCGTTTCACGA
GCGACGGCCAGTCTTTGGTGTNGCCAAGATTCACCAGCGCGTTTTTCAGCCTGACGCACT
CTTCTTCGCCGGTACACAGCGCTTCGGTTNTCAGCACGATCTCCGCGAAATCATCCAGCA
GCACCATGCCCGATTTCTGAATCGNCGACGCCAGCTGCGGGTTGATTTTGGTGTCGCCTTC
CTGCNNGATGCNACTGGCGGTTTANTGCTTCTGCCAGCGCCGTGGCTTTGGTCACCGTGTC
GGATTCCGGCAGAGGCAACGGGCTTGCGTTATTNCAGANAATTTGCGAGCAGTCGACGG
CATAAACNGGTAATTCTGGCGGNTGGTATAGCGTCCNGCAGATGAATATTACACATGCCT
GTGCCGTNATN

BF4
ANAAGAGACAGGTATTACCGTGGCGGTATTCACGCCAGCCGATATACCAGCACATGACC
AGCAATATCAGCCAGCCTGCGATGCCCGCGATACCATTCTGTAACCCGAATTCAATAAGC
CCATAATGACTGTGCGGGATATCAATACGCTGATTATTAGTGTCGTGACGTAAAAGGTAA
TTAAACGCCTCTTTACGCGGCCCCATGCCGAACGGATGTTCTGCGACCAGTTTCCAGCCCT
GATGGAAAAAGCTGGCGCGGCAGCCATTGGAATGATTGATCGGCTTGCCTAAGTCATTAA
CGGGATGTTTTCCGGTGGCTCTGTTGTAACAGAAGCTATTGAAGGAGGCATTCCAGCCCG
TCTCGGCGTCATCTTCCAGACTGCCCCAGCGCGGGTCGGTTTTCCATGAAGTCACCACCA
GCGCGCCTGCCACCACGACGATAGCCAGGAAAATGCTACCCACTTTAATCACATTGCTAC
GCCGCATCTTATGCAGGCTTAGCAGGAGAAAAGTCGAAAAGAGGCTGCCCACAAGGCCG
ATCGTGCCCCAGCGGGTGTCGACCAGTGCGGTGCATAGTAGATTGGCAAGCAACATCAA
AACCAGAACCGGCGATTTTAGCGTCATAAAGCGCTGATGCAGCAGACTGCGCGTCAGTA
ACTCCGCCAGCAAGAAGCCGGTTATCATATTGACCTGGAAGCTCATGCGGGTGCGGTTAA
ACACAATTCGCGTCTCGCCCCAGTGAATGACTCCGTCACGCCAGTAAAGATAAAGCGAGT
CCAGTAAATGGATCAGGATAACGCCCCAGAAACACAGGATCACCGCCGTGAACAGCCTG
GCGGCAGTAAGTGAAGGGAATCTGTCCTGTAAAACCGGCATCAGCAGGATGCCCGAGGT
AAAAAGTAAAACGGGTCTGACCCATTGCCCGTCCCAGGTTCCGATCATTTCTTTAATATC
AGGCGCAACAATAAAACCGTTGATTAAAATAAAGAACGTCAGTAACCAGATGAACAG

51_C4
GATGTGTATAAGAGACAGGGTGAGACCAGGCCGTGGTTTGCGTGGCTCATCGAGGCTTGC
CAGCACCAGCTCCAGCACGCGCTCCGCGACATCGCGATGGCGCTGCGCCACGGCAAGAA
CCGGGCATTGCAGGAAATCGAGCAGTTCGTTATCGCCAAACGTCGCGATAGCGAGCTCTG
ACGGCAAGCGCCCTTCGCGCTTCAGCGTCACGTCCATGACGCCCTGCAATAAAGCGAAAG
AGGTGGTGAACAGCGCCTGTGGCATCGGGTGCGTTTCCAGCCATTTTTCAAACAGCGCGG
CGGCGGCTTCACGCTCGTAGCTGTTGGCATAAAGGAAATGCACTTCGCGCGGGTCATCTT
TCCACGCGGTGCGGAAGCCTTGCTCGCGCAGAAAACTTACCGAGAGCTCCGGCAGCGCG
CCCAGATAGAGGACGGTTTCAGCCGGGAACGTGCGCAGCTCGCTTGCCAGCATTTCCGCA
TCGTCCTGATCGGCACCGACGACGCTGGTGAAGTGTTCGCGATCCAGCGCCCGGTCCAGC
GCCACGATCGGAAATGCGTCGTTCGCCCAGCGCTGGTAAAACGGATGTTCAGGCGGTAA
AGAAGTAGAAACGATGATAGCGTCAACCTGGCGTTGCAGCAGATGCTCAATGCAGCGCA
TTTCGTTATCGGGCTGATCTTCAGAACAGGCGATAAGCAGCTGATATCCGCGCTGGCGCG
CCTGGCGCTCAAGATAGTTGGCGATGCGGGTATAGCTGGTGTTTTCAAGATCGGGGATAA
CAAGGCCAATCGAGCGTGTTCTTCCTGCGCGCAGGCCTGCGGCGACGGCGTTCGGGTGGT

AATTATGCTCACGCACCACCGCCATCACTTTCTCAACGGTTTTATCACTCACACGGNACT

51_C6
GATGTGTATAAGAGACAGCCTTAAAGGAGAGTTCAAGCCAGGCGATATTTTACCTGGCGA
GATGGAGCTGGGTGAACAGTTCGGCGTGAGCCGCACGGCGGTAAGAGAAGCCGTTAAGA
CGCTGACGGCCAAAGGGCTGGTCTTACCGCGCCCGCGTATCGGCACGCGCGTGCTGCCTC
AAAGCCAGTGGAACTTCCTCGATAAAGAACTGCTGACCTGGTGGATGGGCATCGAAACCT
TCACCACGGTGGTTAATCACTTTCTGGTGATGCGTCACAGCCTTGAACCTCAGGCCTGCGC
GCTGGCCGCGCTAAACGGTACTGAAGAACAAAAGCAGCGCTTCAGGAAAACCCTGGATG
AGATGGCCGCGCTCCAGGTCTCTTTTAACCGCGAACGCTGGATAGAAACCGACATGGCCT
ATCATGAACAGATCTATGAAATGAGTGGCAATCCCTTTATGACCGCTTTCGCGAGTCTGTT
CCGCTCCATCTATTACAATTATTTCACGTCGATAACGCACAACCAGGTTATCAAGCCTGAC
ATTCATCAGGCGATTGCGGACGCTATTCTTTCTTCCCAAAGCGAAGAGGCCTATCGCGCC
TGTCAGACGCTTTTGCAGGCGACCGCATCGCAGGAAAAATAACAACAGGATCCGCATGA
CTAAAAAAGCGCGCAGTATGGCGGGCCTGCCGTGGATAGCGGCAATGGCCTTCTTTATGC
AGGCACTGGATGCCACTATCCTCAACACCGCCCTGCCGGCTATCGCTCAAAGTCTTAACC
GCTCGCCGCTGGCGATGCAGTCTGCGATTATCAGCTACACCCTGACGGTCGCGATGTTAA
TTCCCGTCAGCGGCTGGCTGGCAGACCGCTTCGGCACCCGTCGCGTCTTTATGACCGCCGT
CTCCCTGTTCACGCTGGGTTCGCTCGC

69_F1
GGTTGANNTNTGTATANGAGACAGGTTCAGAAAGGGCCGGGCCAGTCCCCGCCATAAAA
CCAGCGGGAAAATACCCACCACCATCGCCACCACGGGCGCCACCAGCACACGGAGCACC
ACGCCCAACGGGGTGATGGTGCCGTCATACACCGCGGGCAGCATCCAGCCAAACGTCGC
CATCATCATGCCGACCAGCCAGAGGCTACGCATCATCGGTCCGTGCGCTTCATTAAGTTT
GTGGTACGTCTGTTGCCACGCGTGGATATCCGCGCCCGCAAAGCGCGGAATAAGCGCAG
GATGGCCAATCGCCAGCTGGTCGGTGAGCCTGCCGCAGGCCTGCCAGTCTTCATCGGTGT
AATCGAGGCCAGCCAGCATGCCTGCAGGTCGACTCTAGAGGATCCCCGGGTACCGAGCTC
GAATTCACTGGCCGTCGTTTTACAACGTCGTGACTGGGAAAACCCTGGCGTTACCCAACT
TAATCGCCTTGCAGCACATCCCCCTTTCGCCAGCTGGCGTAATAGCGAAGAGGCCCGCAC
CGATCGCCCTTCCCAACAGTTGCGCAGCCTGAATGGCGAATGGCGCCTGATGCGGTATTT
TCTCCTTACGCATCTGTGCGGTATTTCACACCGCATATGGTGCACTCTCAGTACAATCTGC
TCTGATGCCGCATAGTTAAGCCAGCCCCGACACCCGCCAACACCCGCTGACGCGCCCTGA
CGGGCTTGTCTGCTCCCGGCATCCGCTTACAGACAAGCTGTGACCGTCTCCGGGAGCTGC
ATGTGTCAGAGGTTTTCACCGTCATCACCGAAACGCGCGAGACGAAAGGGCCTCGTGATA
CGCCTATTTTTATAGGTTAATGTCATGATAATAATGGTTTCTTAGACGTCAGGTGGCACTT
TTCGGGGAAATGTGCGCGGAACCNCTATTTGTTTATTTTTCTAAATACATTCAAATATGTA
TCCGCTCATGAGACAATAACCCTGATAAATGCTTCAATAATATTGAAAAAGGA

1_E1
ATAAGAGACAGCCTATGCGCTATCGCAATACCGGCGTCTCGATGTCCTCGGACGCAAGCG
TTGACGAATATTCCCGTTACCAGGTGGATGGCGACGCGGTAGCCGCCATGAAGGCGGCGC
TTACGTCAGTGCAATGATTTTTGCCCTCAGTTTTTACCGCGCATCATGTCGCCCAATATGA
TGCTTGCTCGTACCAGGCCCCGGTCTTTTGCCGGGGCTTTTTTTATGCCCACGCGCTCCTA
CCAAATCTGGTCACTGTCTTGTCTTTACCGGGCCGCTCAGAGAAGATGCCACGCATCGTC
AGCGTCTTTCGCAGACCTGTAAAGGGAGAGCAGGATGATCGTCGGCATCGATCTCGGTAC
CACCAATAGCCTCGCTGCCGTCTGGCAGGAGGGCAATCCGCGGCTTATCGCTAACGCGCT
GGGCGAATGTCTCACGCCGAGCGTCGTCGGCCTTGATGACGAAGGGCGCGTGCTGGTCGG
CAAAGCCGCCCGCGAGCGTCTGCACACGCATCCGCATCTCACCACCGCACTTTTCAAACG
GCATATGGGCACCGCCTGGACCGTCCGGCTCGGGGGCAAACTGTTCCGCGCTGAAGAACT
CTCGGCGCTGGTTTTAAAAAGCCTGAAAGAAGATATCGAGCGCGCCTGCGGCGAGACGG
TCACCGAAGCGGTGATAAGCGTGCCGGCGTATTTCAGCGATGCCCAGCGTAAAGCCACGC
GTGTCGCGGGCGAACTGGCCGGGCTTCACGTTGAAAAACTGATTAACGAACCGACCGCG
GCGGCGCTGGCTTACGGCCTGCATCGCGGCACAGAAGAGGGCACCTTTCTGGTGTTCGAT
CTCGGCGGCGGCACGTTTGATGTCTCGGTGCTGGAGCTGTTCGAAGGCGTGATGGAAGTA

C

4_G12
TGTATANGAGACAGGTGCCAGGATTGCTGACCGGAGCCAAACTGCGCGCCGCCCTGCATC
ATGCGCAGGAATTGATCGAACTGCATGCCTGCAGGTCGACTCTAGAGGATCCCCGGGTAC
CGAGCTCGAATTCACTGGCCGTCGTTTTACAACGTCGTGACTGGGAAAACCCTGGCGTTA
CCCAACTTAATCGCCTTGCAGCACATCCCCCTTTCGCCAGCTGGCGTAATAGCGAAGAGG
CCCGCACCGATCGCCCTTCCCAACAGTTGCGCAGCCTGAATGGCGAATGGCGCCTGATGC
GGTATTTTCTCCTTACGCATCTGTGCGGTATTTCACACCGCATATGGTGCACTCTCAGTAC
AATCTGCTCTGATGCCGCATAGTTAAGCCAGCCCCGACACCCGCCAACACCCGCTGACGC
GCCCTGACGGGCTTGTCTGCTCCCGGCATCCGCTTACAGACAAGCTGTGACCGTCTCCGG
GAGCTGCATGTGTCAGAGGTTTTCACCGTCATCACCGAAACGCGCGAGACGAAAGGGCCT
CGTGATACGCCTATTTTTATAGGTTAATGTCATGATAATAATGGTTTCTTAGACGTCAGGT
GGCACTTTTCGGGGAAATGTGCGCGGAACCCCTATTTGTTTATTTTTCTAAATACATTCAA
ATATGTATCCGCTCATGAGACAATAACCCTGATAAATGCTTCAATAATATTGAANAAGGA
AGAGTATGAGTATTCAACATTTCCGTGTCGCCCTTATTCCCTTTTTTGCGGCATTTTGCCTT
CCTGTTTTTGCTCACCCAGAAACGCTGGTGAAAGTAAAAGATGCTGAAGATCAGTTGGGT
GCACGAGTGGGTTACATCGAACTGGATCTCAACAGCGGTAAGATCCTTGAGAGTTTTCGC
CCCGAAGAACGTTTTCCAATGATGAGCACTTTTAAAGTTCTGCTATGTGGCGCGGTATTAT
CCCGTATTGACGCCGGGCAANNNCAACTCGGTCGCCGCATANNNTA

21_G1
GAGACAGGCATAANGGTATCATCCAGATATTCATCTAATTGCTCAANNAGAGCTGCGTCA
TCTTCATGCTTAATTTTATAATTCAGGCCGAAGCTGGCAATATGCTCAATCAAGTCGTTGA
GTTGCAGGTTAACGGCAGAAGTGGGGTCGTTAACCCAGCCATGCTGGTTTTCACCGAGCG
TGGCAAGGCAATCATGATACAGGCTTTCGCAAAGAAATTTAAGCTGCGCTATATCATGCC
GTTTTGGTGAGTATTCGTCCATAACGTATCCCCTTTTTAGCGAAAAATTTAGTCAACAGC
ACCGCAGGGGCAGATACACAACCAGTGTCTGGAGCCGTGGCGCTCCATTTTTAATCTACT
GTTACTATAAAACACCCTGGGGATTTTTTCCTGGCACGATTACGAAAAATTACATTTCCGC
GGTTAATCGCACATCGGCGCAGACTCTTTTACGCGAATCAATAAAGGCAAGGCACGCAG
ACGCATTCAGAAAGAAAACTTAACCTTTAAGTCTTAGTTTAACCTTAGTTTAAAACGTTTA
TTTATTCACGCCTGAAGAATATTCTGTAATAAAGGTTTTTCTGCATCAGATTAATCCCAA
TAAAATATTTATAGCGAAAAAAAGAGAAACGAAAGGNNCNAGTGATGTTTTTTGNNNG
AAGCGTTAAAANNCGTATGCAGGGAAGGGGTAAAAAAAAGGCCNCATCTNNNGCCTTTC
TT

24_H4
ATGTGTATAAGAGACAGTGTGACATCTACCAGGAAGAGGTCAACGTTGTGGAGCCTCTGT
TCTCCAACTTCGGCGGTCGCTCTTCTTTTGGCGGGCAAATCATCACGGTGAAATGTTTCGA
GGACAACGGGTTGCTGTATGAGCTGCTCGAAGAAAATGGCCGCGGACGCGTTTTACTGGT
CGATGGCGGCGGTTCGGTACGCCGCGCCCTGATCGATGCGGAACTGGCGCGCCTTGCGGT
GCAGAATGAATGGGAAGGGCTTGTCGTCTACGGCGCGGTGCGTCAGGTGGACGATCTGG
CGGAGCTGGATGTCGGCATCCAGGCGCTGGCGGCGATCCCGGTGGGTGCTGCGGGCGAA
GGCATCGGCGAAAGCGACGTGCGCGTCAATTTCGGCGGCGTGACCTTCTTCTCCGGCGAC
CACCTCTACGCCGACAATACCGGTATTATCCTTTCTGAAGATCCTCTGGATATCGAGTAAC
AAAAACGGGCGCCCTGAGCGCCCGTTCTTTTTGGTTCAAAGTGCGGTAATCACACTTCTTC
CATTTTTCCCAGCAGCGCCTGCAGGCGCTCCTGCCAGACATGCTGCTGTTCGCGCAGCTG
GTGGTTTTCCTGCTCCAGCGCTTCGCGGCTGCCCTGGGCATTCTGAACTTCCTGAGCAAGT
TGATTGTTCTTGTCTTTCAGCTCTTCGATTTCCATCTGCAGCAGGGTGATAGTATCAACAG
CCTGCTGAACTTTGGCTTCCAGTTTCTCAAACACTTCAAATGACATCGTCCTACCTCTCCT
GAATTGCAAGGCGTTGATGGATAACTATCCTCGTCCCGGTGCCCGGCGACGCCTTAAATT
AATCTGTGCGCACAACACTAGTCCCGATTGTATGAAGCCCTTGTGCCCCTGT

37_A11
NCAGGGCGTANNGCGTGCGGCCCTGCTTCTCAGACAGCCACAGCACNTNGTTATCGATTT
CTTCGTCCGGAATGCCATCGATGATGCCATGTATCAAATCGAGATCTTCACTGGCCCAGG
GCTTTCCGCTACGCGGAAACACCAGGTCGGGTTCACGTGGTTTTTAGGCGGCTTTTCGGC
CTTCGCCTGTTTGCGTGCTTCTTTTTCTGCCGTAGCCTGACGTATCCGCTCGTTAAGACCTC
CGATCGTGGCCACGGTCATGACCATATTTTCACGCTGTTCCCGCGTCAGCAGACCATCCTC
ACAGATAAGATCATTGAGGGTGGAGAGCAGCGTGTTAATCCGCGTCTCCGTGAGTTTTAC
GCCCATATCAGCCTGTCCTTTTCATTTACCGGCTGCCCTCAGCGGCAGCTCGGGTTATTTC
GTCCAGTCTTCCAGCATCAGTCCCGGAACACGTTCAAATTCACGCACATTATTGGTAACC
AGTACGGCGCCGGCTGCAATGGCATGCCTGCAGGTCGACTCTAGAGGATCCCCGGGTACC
GAGCTCGAATTCACTGGCCGTCGTTTTACAACGTCGTGACTGGGAAAACCCTGGCGTTAC
CCAACTTAATCGCCTTGCAGCACATCCCCCTTTCGCCAGCTGGCGTAATAGCGAAGANGC
CCGCACCGATCGCCCTTCCCAACAGTTGCGCAGCCTGAATGGCGAATGGCGCCTGATGCG
GTATTTTCTCCTTACGCATCTGTGCGGTATTTCACACCGCATATGGTGCACTCTCAGTACA
ATCTGCTCTGATGCCGCATAGTTAANCCAGCCCCGANACCCGCCAANACCCGCTGNNNCG
CCCTGACGGGCTTGNNTGCTCCCGGCATCCGCTTANAGACAAGCTGNGACCGTCTCCGGG
ANCTGCATGNGTCNNAAGGTTTTCNCCGTCATCNCCGAAACGCGCGNNACGAAAGGGCC
TCGTGANACGCCTATTTTTANAGGTTAATGTCATGATAATAATGGTT

BF4 insert (f. complementation)
TAAGAAGGGTTAGTTGACGCTTCTGGCGGCGTGCGCAGGGCGCAAATCCCAATTAACAGC
CCCGTCAGCATCATATACTGCTCCAGATAATGGTCTTTAAGGTTATGGTCAACCAGGGTA
CGAGAGAAGAAACCAATCGTAAACAAGAGCAAGAAAAGCCCTGGCATCGTATTACCGTG
GCGGTATTCACGCCAGCCGATATACCAGCACATGACCAGCAATATCAGCCAGCCTGCGAT
GCCCGCGATACCATTCTGTAACCCGAATTCAATAAGCCCATAATGACTGTGCGGGATATC
AATACGCTGATTATTAGTGTCGTGACGTAAAAGGTAATTAAACGCCTCTTTACGCGGCCC
CATGCCGAACGGATGTTCTGCGACCAGTTTCCAGCCCTGATGGAAAAAGCTGGCGCGGCA
GCCATTGGAATGATTGATCGGCTTGCCTAAGTCATTAACGGGATGTTTTCCGGTGGCTCTG
TTGTAACAGAAGCTATTGAGGGAGGCATTCCAGCCCGTCTCGGCGTCATCTTCCAGACTG
CCCCAGCGCGGGTCGGTTTTCCATGAAGTCACCACCAGCGCGCCTGCCACCACGACGATA
GCCAGGAAAATGCTACCCACTTTAATCACATTGCTACGCCGCATCTTATGCAGGCTTAGC
AGGAGAAAAGTCGAAAAGAGGCTGCCCACAAGGCCGATCGTGCCCCAGCGGGTGTCGAC
CAGTGCGGTGCATAGTAGATTGGCAAGCAACATCAAAACCAGAACCGGCGATTTTAGCG
TCATAAAGCGCTGATGCAGCAGACTGCGCGTCAGTAACTCCGCCAGCAAGAAGCCGGTT
ATCATATTGACCTGGAAGCTCATGCGGGTGCGGTTAAACACAATTCGCGTCTCGCCCCAG
TGAATGACTCCGTCACGCCAGTAAAGATAAAGCGAGTCCAGTAAATGGATCAGGATAAC
GCCCCAGAAACACAGGATCACCGCCGTGAACAGCCTGGCGGCAGTAAGTGAAGGGAATC
TGTCCTGTAAAACCGGCATCAGCAGGATGCCCGAGGTAAAAAGTAAAACGGGTCTGACC
CATTGCCCATCCCAGGTTCCGATCATTTCTTTAATATCAGGCGCAACAATAAAACCGTTGA
TTAAAATAAAGAACGTCAGTAACCAGATGAACAGCATCACCGGTTTGATTTTAGGTATGT
TTAAATGCTGACGAGCTGAAGACGACGTAAAAAACAGGCCAACAGTGAGCGCAATAGAA
AGATAGATAAAGACGTTGCGATGGAAAGGTAACATTCCATTCGGAATAGGCCATACAAA
GCAAAGCGCCAGTACGGATACGAATAAAAGCCCGGACAAAGCATTACGTTTAGTGTTCA
TAAAAATTAACAACTTGTAAGGACAGTTTGCTTAACATGTGGTCTTAAAAATTCAGAAAT
ATCTCGTAAGACTCTGCGAAAAAACAATAAATTGCACGGCAAAGTAGCACAGATAATAT
CTATAAAGAAGGATAATGTTTGGATCCCCGGGTACCGAGCTCGAATTTAATACGACTCAC
TATAGGGAATTCCTGTTATAAAAAAAGGATCAATTTTGAACTCTCTCCCAAAGTTGATCC
CTTAACGATTTAGAAATCCCTTTGAGAATGTTTATATACATTCAAGGTAACCAGCCAACT
AATGACAATGATTCCTGAAAAAAGT
